# Lung sealant and morbidity after pleural decortication: a prospective randomized, blinded study

**DOI:** 10.1186/1749-8090-5-45

**Published:** 2010-05-28

**Authors:** Luca Bertolaccini, Paraskevas Lybéris, Emilpaolo Manno

**Affiliations:** 1 Thoracic Surgery Unit, S. Croce Hospital, Cuneo, Italy; 2 Division of General Surgery, Maria Vittoria Hospital, Turin, Italy; 3 Intensive Care Unit, Maria Vittoria Hospital, Turin, Italy

## Abstract

**Objectives:**

Prolonged postoperative air leaks (AL) are a major cause of morbidity. Aim of this work was evaluating use of a Lung Sealant System (Pleuraseal™, Covidien, Mansfield, MA, U.S.A.) in pleural decortications for empyema thoracis.

**Methods:**

From January 2008 to December 2008, 46 consecutive patients received pleural decortications for empyema thoracis. Post-procedural and malignancy-related empyemas were excluded. After hydro-pneumatic test and surgical correction of AL (until satisfaction), patients were assigned (23 per group) to Control or Sealant group. Control group underwent no additional interventions. In Sealant group, lung sealant was applied over AL areas. Following variables were measured daily: patients with AL; time to chest drainage (CD) removal; CD drainage volume at removal, postoperative length of hospital stay, postoperative C-reactive protein (CRP), and leukocyte counts. Personnel recording parameters were blinded to intervention. Two-tailed t-tests (normally distributed data) or Mann - Whitney U-test (not-normally distributed data) were used for evaluating significance of differences between group means or medians. Significance of any proportional differences in attributes were evaluated using Fisher's Exact Test. Statistical analysis was carried out using R-software (version 2.8.1).

**Results:**

Groups were similar regarding demographic and baseline characteristics. No patients were withdrawn from study; no adverse effects were recorded. There were no significative differences on CRP and leukocyte levels between two groups. Compared with the Control group, in Sealant group significantly fewer patients had AL (30 versus 78%, *p = 0.012*), and drains were inserted for a shorter time (medians, 3 versus 5 days, *p = 0.05*). Postoperative hospitalization time was shorter in Sealant group than in control group, but difference was not significant (0.7 days, *p = 0.121*).

**Conclusions:**

Pleuraseal™ Lung Sealant System significantly reduces AL following pleural decortications for empyema and, despite of not-increased infectious indexes, is suitable for routinely use, even in procedures with contaminated pleura.

## Introduction

Prolonged parenchymal air leak is the most common complication after lung resection [1]. Prolonged air leak lead to prolonged chest tube drainage time, associated with pain and immobilization that puts the patients at an increased risk for development of infections and bronco-pleural fistulae and, consequently, a prolonged hospital stay, which increases healthcare costs [2]. A variety of complementary natural and synthetic materials have been tried to overcome such complications including fibrin sealants, collagen fleece, and synthetic glues [3]. Nevertheless, they can be very helpful in situations where air leakage cannot be assured by classic means [4]. Additional techniques included the application of a number of products such as fibrin glue [5-7], synthetic sealants [8-10] and fleece bound sealants [11]. However, major criticisms elicited by currently available studies include lack of a precise methodology, usually limited numerosity, and presence of significant confounding factors (i.e. postoperative air leak assessment) [12,13]. Empyema thoracis remains a significant cause of morbidity and even mortality in modern thoracic practice [14]. In surgical decortication, the risk of air leak was increased, but most of sealant cannot be use in infected pleural space [15]. We have tested the application of a lung synthetic sealant to evaluate the safety and efficacy of this surgical sealant for the treatment of parenchymal air leaks occurring after pleural decortication within the setting of a prospective randomized blinded trial.

## Materials and methods

This prospective randomized blinded study was carried out between January 2008 and December 2008. Forty-six consecutive adult patients with pleural empyema that received pleural decortication for empyema thoracis were enrolled. Post-procedural and malignancy-related empyemas were excluded. Three skilled Thoracic Surgeons carried out pleural decortication using standard techniques. At the end of the operation, before closing the chest, the lung was ventilated under positive-end expiratory pressure and warm saline was installed into the chest cavity to test air leaks. The operating surgeon corrected any leaks surgically, until satisfied. The patients were, then, assigned (23 per group) to either the Control Group or Sealant Group by opening a sealed envelope that contained the randomization code (allocated by a computer generated random sequence, http://www.random.org/premium/?mode=advanced). Patients assigned to the Control Group underwent no additional interventions. In patients assigned to the Sealant Group, lung sealant (Pleuraseal™, Covidien, Mansfield MA, U.S.A.) was applied over the entire areas of air leaks with the lung partially inflated (65 - 75%). After the application, the lung was completely ventilated and the chest closed with two thoracic drains (32 F) set at suction at -20 cm H_2_O. Following variables were measured and recorded daily, until discharged from hospital: rate of air leakage on the day of the operation, daily rate of air leak until removal of chest drains; time to chest drainage removal; chest drainage volume (bleeding/exudation) at removal of chest tube, postoperative length of hospital stay, postoperative C-reactive protein, and leukocyte counts. The rate of air leakage was assessed by a mechanical suction pump (Drentech P.A.L.M., Redax S.r.l., Mirandola (MO), Italy) and was expressed in liters per hour. Personnel recording parameters were blinded to the group assignment. Adverse events were monitored throughout the patients hospital stay. Postoperative air leakage, chest tube drainage and hospitalization time were used as indicators of postoperative morbidity.

Data for each variable and within each randomized group were tested for significant deviation from a normal distribution using the Kolmogorov-Smirnov test for a given cumulative distribution function F(x) (defined as  where  is the indicator function, equal to 1 if *X*_*i *_≤ *x *and equal to 0 otherwise). Two-tailed t-tests (normally distributed data) or Wilcoxon-Mann-Whitney U-test (non-normally distributed data) were used for evaluating the significance of differences between group means or medians, as appropriate. The significance of any proportional differences in attributes (e.g. air leakage present or absent) were evaluated using the Fisher's Exact Test (two groups). Statistical analysis was carried out using R-software (version 2.8.1), a free software environment for statistical computing and graphics that compiles and runs on a wide variety of UNIX platforms, Windows and MacOS http://www.r-project.org/.

## Results

Both groups were similar with regard to demographic and baseline characteristics (Table 1). The mean age for the total study population was 54 years (standard deviation: 11 years). Thirty-four patients (73.9%) were smokers. There was no statistical significant difference with regard to their forced expiratory flow in one second (FEV_1_). Patients' general conditions were considered normal risks for decortication for empyema thoracis. No patients with bullous disease or emphysema were found. No patients were withdrawn from the study and no adverse effects were recorded during the study. Results for each of the performance variables for both randomized groups are summarized in Table 2. Sealant Group was associated with a mean lower total drainage volume compared with the Control Group (534 ml ± 149 ml vs. 873 ml ± 257 ml). This difference was highly statistically significant *(p < 0.001)*. Number of patients without postoperative air leak was significantly greater (7 patients, 30%) in the Sealant Group compared to the Control Group (18 patients, 78%), *p < 0.012*. Although median durations of chest drainage and hospitalization times were shorter in the Sealant Group compared with Control Groups (3 days (quartiles 1.5) vs. 5 days (quartiles 1.4), *p < 0.05*), the postoperative hospitalization were not statistically significant (4 days (quartiles 4.5) vs. 4.5 days (quartiles 4.7), *p < 0.121*). Postoperative leukocyte levels were similar in both groups (10.9 10^3 ^g/l ± 3.2 10^3 ^g/l vs. 10.7 10^3 ^g/l ± 3.1 10^3 ^g/l, *p < 0.95*). Postoperative C Reactive Protein levels were not statistically different between two groups (11.3 g/l ± 2.3 g/l vs. 10.9 g/l ± 1.7 g/l, *p < 0.97*).

**Table 1:** Demographic and baseline characteristics.

Variable	Sealant Group	Control Group	*p value*
Age: years ± SD	53 (12)	55 (10)	*0.68*

Male: n (%)	11 (48%)	12 (52%)	*0.55*

Female: n (%)	9 (39%)	14 (61%)	*0.63*

Smokers: n (%)	18 (78%)	16 (70%)	*1.00*

Predicted FEV_1_: % ± SD	76 (19)	85 (18)	*0.09*

Predicted CO-diff: % ± SD	76 (10)	81 (21)	*0.31*

FEV_1_: forced expiratory volume in one second; CO-diff: carbon monoxide difference.

**Table 2:** Results.

Variable	Sealant Group	Control Group	*p value*
Patients (%) with air leak	7 (30%)	18 (78%)	*0.012*

Total drainage volume (ml): mean ± SD	534 ± 149	873 ± 257	*<0.001*

Days with drain: median (quartiles)	3 (1.5)	5 (1.4)	0.05 *

Postoperative hospitalization (days): median (quartiles)	4 (4.5)	4.5 (4.7)	0.121 *

Postoperative leukocyte level (10^3 ^g/l) ± SD	10.9 ± 3.2	10.7 ± 3.1	*0.95*

Postoperative C-reactive protein level (g/l) ± SD	11.3 ± 2.3	10.9 ± 1.7	*0.97*

SD, standard deviation.

* Data was not normally distributed and was analyzed by non-parametric statistics (Wilcoxon-Mann-Whitney U-test; exact significance shown)

## Discussion

Intra-operative air leaks after standard pleural decortication are reported ranging between 48% and 70% in main series [16,17]. For this reason, increasing requirements for new sealant products has stimulated active industrial research and clinical experimentation. Recently, a meta-analysis has showed the absence of a definitive advantage from using sealants in pulmonary surgery when the end-points are the reduction of in-hospital length of stay and postoperative morbidity [17]. Current clinical experimentation in this field has indicated that sealant materials have to display a number of characteristics. They need to be adherent to pressure of inflated lung, but also elastic and unaltered by intrapleural fluids; they also need to be non-irritating, systematically non-toxic, and without antigenicity [12]. Pleuraseal™ (Covidien, Mansfield MA, U.S.A.) sealant, whose basic mechanism is the rapid formation of a biocompatible hydro-gel that firmly adheres to the tissue, has many of these characteristics. Application of this sealant has allowed significant reduction of proportion of patients showing post-operative air leaks. Furthermore, among the patients who received sealant therapy but showed postoperative air leaks, the duration of air leaks was significantly shorter, if compared with the standard care group. Despite the clinical advantage obtained by the earlier air leak cessation, a statistically significant reduction in the duration of the hospital stay was not found in patients receiving sealant application; although they showed a favorable trend towards shorter hospitalization, (air leakage is not the only major reason for postoperative hospitalization). We have not observed any specific complication related to the use of the sealant and postoperative morbidity was similar in the two groups. In particular, we do not found increase in the postoperative levels of leukocyte and CRP in the Sealant Group.

The application of Pleuraseal™ sealant in this randomized study proved safe and effective in reducing air leaks occurring after pleural decortications for empyema thoracis, in shortening the duration of air leak with a trend towards a shorter postoperative hospital stay, and despite of not-increased infectious indexes, is suitable for routinely use, even in procedures with contaminated pleura.

## Competing interests

The authors declare that they have no competing interests.

## Authors' contributions

LB conceived of the study in Maria Vittoria Hospital (where worked, as Consultant, until 2009), performed the statistical analysis and participated in design and coordination of the study. PL participated in data collection. EM participated in design and coordination of the study. All authors read and approved the final manuscript.
